# Reproductive healthcare utilization in urban poor settlements of Delhi: Baseline survey of ANCHUL (Ante Natal and Child Health care in Urban Slums) project

**DOI:** 10.1186/s12884-015-0635-8

**Published:** 2015-09-08

**Authors:** Niveditha Devasenapathy, Suparna Ghosh Jerath, Elizebeth Allen, Saket Sharma, Anuraj H. Shankar, Sanjay Zodpey

**Affiliations:** Indian Institute of Public Health, Delhi, Public Health Foundation of India, Plot No. 47, Sector 44, Institutional Area, Gurgaon, 122002 India; Department of Medical Statistics and Faculty of Epidemiology and Population Health Department, London School of Hygiene and Tropical Medicine, Keppel Street, London, UK; Department of Nutrition, Harvard School of Public Health, 665 Huntington Ave, Boston, MA 02115 USA

## Abstract

**Background:**

Disparity in utilization of reproductive healthcare services between the urban poor and the urban non-poor households in the developing nations is well known. However, disparity may also exist within urban poor households. Our objective was to document the extent of disparity in reproductive healthcare utilization among the urban poor and to identify the socio-demographic determinants of underutilization with a view to characterizing this vulnerable subpopulation.

**Methods:**

A survey of 16,221 households was conducted in 39 clusters from two large urban poor settlements in Delhi. From 13,451 consenting households, socio-demographic data and information on births, maternal and child deaths within the previous year was collected. Details of antenatal care (ANC) was collected from 597 pregnant women. Information on ANC and postnatal care was also obtained from 596 recently delivered (within six months) mothers. All data were captured electronically using a customized and validated smart phone application. Households were categorized into quintiles of socio-economic position (SEP) based on dwelling characteristics and possession of durable assets using principal component analysis. Potential socio-demographic determinants of reproductive healthcare utilization were examined using random effects logistic regression.

**Results:**

The prevalence of facility based birthing was 77 % (*n* = 596 mothers). Of the 596 recently delivered mothers only 70 % had an ANC registration card, 46.3 % had ANC in their first trimester, 46 % had visited a facility within 4 weeks post-delivery and 27 % were using modern contraceptive methods. Low socio-economic position was the most important predictor of underutilization with a clear gradient across SEP quintiles. Compared to the poorest, the least poor women were more likely to be registered for ANC (OR 1.96, 95 %CI 0.95-4.15) and more likely to have made ≥ 4 ANC visits (OR 5.86, 95 %CI 2.82-12.19). They were more likely to have given birth in a facility (OR 4.87, 95 %CI 2.12-11.16), to have visited a hospital within one month of childbirth (OR 3.18, 95 %CI 1.62-6.26). In general, government funded health insurance and conditional cash transfers schemes were underutilized in this community.

**Conclusion:**

The poorest segment of the urban poor population utilizes reproductive healthcare facilities the least. Strategies to improve access and utilization of healthcare services among the poorest of the poor may be necessary to achieve universal health coverage.

**Electronic supplementary material:**

The online version of this article (doi:10.1186/s12884-015-0635-8) contains supplementary material, which is available to authorized users.

## Background

There is considerable disparity in availability, accessibility and affordability of reproductive and child health services between the rich and poor living in urban settlements in developing countries [1–3].

A meta-analysis of Demographic Health Surveys (DHS) from 31 developing nations has shown that, the odds of having a skilled attendant at delivery was 94 % lower for women in poorest wealth quintile and five times higher in women with complete primary education [4]. Among women with complete education, the likelihood of using modern contraception and attending four or more Antenatal care (ANC) visits were 2.01 and 2.89 times higher respectively, as compared to those with less education [4]. Across the Indian subcontinent on an average the wealthiest quintile have twice the coverage of Maternal Neonatal and Child Health (MNCH) care services as compared to the poorest [5]. Among the Indian urban population a secondary analysis of National and Family Health Survey-3 (NFHS 3) data comparing urban poor with non-poor population showed higher utilization amongst the urban non-poor (the odds of ANC being 1.48, medical assistance in delivery 2.16 and use of modern contraceptives 1.34 times higher in urban non-poor) [6]. This disparity and its potential causes; an interplay of economic, social and political factors are well recognized [7].

However, the widespread variation in access to healthcare *within* segments of the urban poor population is less well appreciated. Some studies have found that the ultra-poor sub-population within the urban poor have the least access to healthcare due to extreme poverty, lack of awareness and social exclusion [8–10]. The determinants of poor access and utilization are likely to vary with differing local contexts both within and across countries [2]. Identifying the inhibitory factors for access to healthcare specific to a population is crucial to ensure uniform coverage and uptake of health programmes, particularly those aiming at Universal Health Coverage (UHC) [11]. While the Government of India appropriately aims at *“Ensuring equitable access for all Indian citizens, regardless of income level, social status, gender, caste or religion, to affordable, accountable, appropriate health services of assured quality..”,* through its proposed UHC program, [12] there is little documentation of the factors and their magnitude responsible for the disparity in access within urban poor populations in India. There is need to develop context specific strategies to identify this deprived population requiring focussed attention. It has been recognized that “all slums are not equal” and tools have been developed for rapid assessment of vulnerability of clusters [13, 14]. Similarly within a settlement the socio-economic position of households vary widely and there is need to identify the most vulnerable.

Our objective, was thus to assess the vulnerability of households within the urban poor communities of Delhi with respect to utilization of reproductive health care. Further, we also explored the various socio-demographic determinants at community and household level that influence access to reproductive health care in pregnant and recently delivered mothers.

## Methods

This report is based on the information generated from the baseline survey of 16,221 households as part of the larger ANCHUL (Ante Natal and Child Health care in Urban Slums) project. ANCHUL is a quasi-experimental implementation research project aimed at assessing the effectiveness of a complex intervention in improving utilization of maternal and child health services in urban poor settlements of Delhi. We documented the extent of reproductive healthcare utilization and explored associations between socio- demographic characteristics and non-utilization of services with a view to characterize the most vulnerable subpopulation within a settlement.

### Setting

National capital of India, Delhi comprises of 11 administrative districts. One in every five resident of Delhi lives in slums and nearly half in other urban poor habitations like unauthorized and resettlement colonies [15]. Health services are offered both by the public and private providers. Public health service administration is the joint responsibility of both the central and state government health departments and is offered through Primary Urban Health Centres (PUHCs), Maternity and Child Welfare centres, Maternity homes and Referral hospitals. Our study was a cross sectional survey in the South-East district of Delhi. A total of 22 PUHC’s, three Maternity and Child Welfare centres, one Maternity home and a referral hospital caters the South East district, which has an approximate population of 1.5 million. Many of the urban poor settlements have well demarcated administrative boundaries and are catered by a PUHC.

### Sampling

For the purpose of ANCHUL project, two such urban poor settlements, each catered by a PUHC in South-East district were purposively selected by the Delhi State Health Mission, Government of Delhi. The study area (viz. Sangam Vihar and Lal Kuan) are further divided into 13 administrative blocks of varying sizes. For the purpose of deploying community health workers (Accredited Social Health Activist, ASHA), the entire area was demarcated into smaller clusters comprising of approximately 400 households. This demarcation was a combined exercise of the study team and the medical officer in-charge of the PUHCs which resulted in 39 clusters. During the cluster demarcation procedure a detailed lane mapping was done and all households were listed. The study sample included all consenting households from this list. This cross-sectional survey was conducted between, October 2013 and February 2014.

### Data collection procedure

**Fig. 1:**
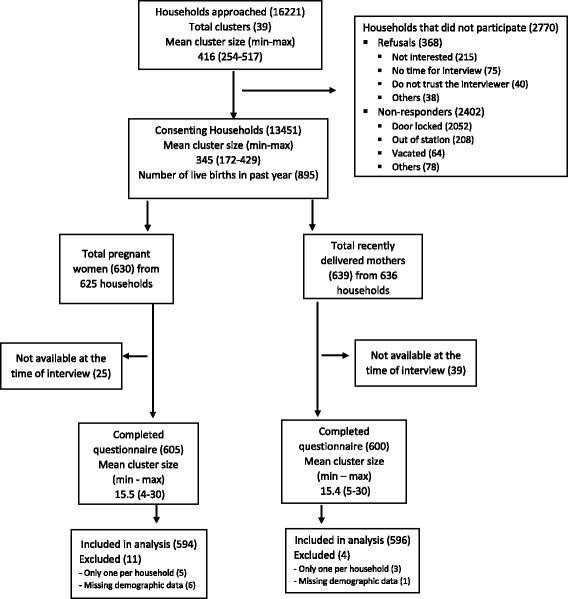
STROBE flow chart of the survey population

All data were collected by field workers using smart phones. The e-forms in local language (Hindi) were developed using the CommCare HQ [16] a mobile app by DIMAGI [17]. This data capture tool with extensive in-built checks was validated and field workers were trained in the use of this e-data collection app (See Additional file 1 for further details of data collection procedure). Consent was obtained from the block representatives before start of the survey. Written informed consent was obtained from the respondent who was a member of the household or any family member who was above 18 years of age. Questions on family member details, household assets, basic facilities within the household, information on births and any maternal and child deaths in the past year were collected from all consenting households. Houses which were locked were visited 3 times including a visit in the weekend, before being categorized as non-responders and in case of refusals the reason for refusal was documented. If there was a pregnant women within the household (self-declared) then information on ANC was collected. In households with mothers who had delivered in the past 6 months information on number of antenatal visits, place of birthing, birth weight of the neonate, post-partum visit to hospital, breast feeding practices and contraception practice currently followed by the woman were collected. Systematic and random checks were done in the field to ensure coverage of all households and accuracy of data by field supervisors. Figure 1, of Additional file 1 describes the data management procedures followed in this survey. The study protocol was approved by the Institutional ethics committees of Public Health Foundation of India, WHO Geneva, All India Institute of Medical Sciences, New Delhi, and Harvard School of Public Health, Boston, USA.

### Exposure and outcome variables

Exposure variables were measured at cluster, household and individual levels. The cluster level variables were a composite vulnerability score and distance of the cluster from PUHC in kms. The household level factors were religion, caste, type of family (nuclear/joint), family size (discrete variable) and a composite score for socio-economic position of the households. The individual level variables considered were literacy (literate by formal or informal education/Illiterate), parity (primi vs. multi).

The vulnerability score (0–10) was calculated using the scoring system proposed by Osrin et al. [13] which included information on hazardous location of clusters, type of housing, percentage of households with metered electricity, piped water, private toilets and home ownership. The presence of garbage dumps, water bodies, railway tracks, distance of the cluster from the PUHC, number of clinics and pharmacies, schools and *Anganwadi* centres (Maternal and Child Community centre) in the study area were also recorded.

Socio-Economic Position (SEP): This was derived from dwelling characteristics and household assets using principal component analysis (PCA) [18]. Details are in Additional file 2.

The outcomes were process indicators of healthcare utilization among pregnant women (PW) and recently delivered mothers (RDM), namely, possession of ANC card, first ANC visit within first trimester, ANC visits to hospital, place of childbirth, post-partum hospital visit, use of modern contraceptive methods and possession of entitlement cards. The definitions are provided in Table 1.

**Table 1:** Definition of outcome indicators with sample size used in the analysis

Outcome indicators	Definition	Sample population (n)
Possession of antenatal care (ANC) card,	Pregnant women reporting possession of a a ANC card/prescription issued by a health care facility (Public /private)	Pregnant women (594)
Number of ANC visits to hospital	(1) Adequate (4 and more)/no visits or inadequate (0–3)	Recently delivered mothers (596)
	(2) No ANC visits/Some ANC	(Does not include those who had miscarriage)
ANC registration in first trimester	First ANC visit to a hospital within 3 months of pregnancy	Recently delivered mothers who had some ANC (417)
Place of childbirth	Facility or Home	Recently delivered mothers (596)
Post-partum hospital visit	Any visit to hospital within 1 month from the time of childbirth irrespective of the place of delivery	Recently delivered mothers (596)
Modern contraceptive use	All contraceptive methods adopted by the couple except for natural methods like coitus interruptus or rhythm method	Recently delivered mothers (596)
Possession of entitlement cards.	-Ration cards (used to get subsidised food from Public Distribution System)	Pregnant women and Recently delivered mothers (1183)
	-Below poverty line cards (BPL)	
	-National health insurance smart card (Rashtriya Swasthya Bima Yojana, RSBY)	
	-National unique identity card (Aadhar card, http://uidai.gov.in/) were all considered as entitlement cards.	

### Sample size

The sample size was calculated for the main objective of the ANCHUL project for the outcome of institutional deliveries. The prevalence of institutional deliveries was found to be 33.4 % among the urban poor in Delhi as per the NFHS-3 survey data [19]. However, the formative phase of the ANCHUL study provided a prevalence estimate of 48 % and between cluster coefficient of variation (k) for this variable was 0.22 [20]. Considering the current crude birth rate in India as 21 per 1000 mid-year population, there would be approximately 40 childbirths per year per cluster with at least 400 households (ASHA coverage area). With these assumptions a total of 39 clusters (15,600Households and 1560 births assuming equal cluster size) would give 90 % power to detect a 30 % relative increase in institutional deliveries in the intervention arm compared to the control arm, with two sided alpha set at 0.05 and a coefficient of variation for this outcome assumed to be at 0.20 [21]. In this baseline survey of ANCHUL project, we covered approximately 16,000 households. Sample size was not calculated for the outcomes reported in this report.

### Statistical analysis

Continuous variables were summarized as means or medians with SD or IQR respectively and categorical variables were presented as percentages along with the frequency for descriptive purposes. The prevalence of all the outcome indicators were presented with a 95 % CI that allowed for clustering of households belonging to same ASHA service area. There was little missing data, therefore only households with complete information for all covariates for a given outcome were included in the analysis. To explore the determinants for each of the outcomes, a random intercept model using logistic regression was used to account for clustering. Variables for inclusion in the model were determined *a priori* based on published literature [1, 20, 8, 22, 23] and our formative phase findings. All analyses included cluster level factors: cluster vulnerability score, distance from PUHC in kms (continuous scale), Household level factors: family size (upto 5/>5 members), religion (muslim/non-muslim), caste (SC/ST, OBC, General), family type (nuclear/extended), age of the mother in years (this variable was divided by 3 for easy interpretation of the coefficient), literacy of the woman (literate/illiterate), parity (primi vs multi). If in a household there was more than one woman who had delivered in the past year, only one of the randomly chosen women contributed to the analysis to avoid clustering at the household level. No interactions were expected *a priori*. Maximum likelihood method was used for parameter estimation and adjusted effects are presented as an OR with 95 % CI. For fixed effects we reported Wald test *p*-value. Likelihood ratio test was used to test for random effects. Only cluster was added in the random component of the model. No adjustment was done to account for multiple analyses. Intra-cluster correlation coefficients (ICC) and Median OR, calculated as mentioned in Merlo et al., are reported for all the key outcome variables [24]. For the cluster level factors that were close to at 0.05 alpha cut-off, we also reported the interval OR-80(IOR-80). If the IOR-80 included 1 then it indicates that the cluster level variable is not that important when compared to the residual cluster level heterogeneity [24]. All analyses were done using STATA 13 using the .melogit function [25].

## Results

Of the 16,221 households approached in the study area, 13,451 agreed to participate in the survey. A total of 368 households (2 %) refused to take part and, 2402 (14.8 %) could not be interviewed as the houses were locked on three occasions. The mean cluster size was 416 households (minimum 254 and maximum 517). At the time of the survey, 630 women were pregnant (self-declared) and 639 had recently delivered (i.e. in the past 6 months). Of these, 600 PW and 605 RDM gave information about their pregnancy and delivery of which only 594 PW and 596 RDM were included in the analysis (Fig. 1). The characteristics of the study population (at cluster, household and individual level) are presented in Table 2. The survey indicated that the study area included were mature, densely populated and relatively stable settlements with poor drainage facilities. There were a total of 880 live births in the past one year, 3 maternal deaths and 43 child deaths in the past year.

**Table 2:** Cluster, Household level and characteristics of currently pregnant women (PW) and recently delivered mothers (RDM)

Cluster level	*N* = 39 clusters
Mean cluster size (range) by households present	416 (254, 517)
Mean cluster size (range) by population	1584.3 (704, 2099)
Median vulnerability score (IQR)	2 (2, 4)
At least 1 Anganwadi centre (%)	35 (89.7)
At least 1 NGO present (%)	19 (48.7)
At least 1 clinic (registered/unregistered)	30 (76.9)
At least one pharmacy (%)	13 (33.3)
Mean distance (km) to PUHC(SD)	0.66 (0.32)
Household level	N = 13451 households
ᅟMean family size (SD)	4.58 (2.02)
ᅟConcrete household structure (%)	11256 (83.7)
ᅟPiped water supply within household (%)	10944 (81.4)
ᅟMetered Electricity supply (%)	13157 (97)
ᅟClosed drainage (%)	2730 (20.3)
ᅟToilet within household (%)	11720 (87)
Religion (%)	
Hindu and other religion	11687 (86.9)
Muslim	764 (13.1)
Caste (%)	
General	5590 (41.6)
Scheduled caste/tribe	5135 (38.2)
Backward class	2726 (20.1)
Family type (%)	
Nuclear	10723 (79.7)
Joint	2351 (17.5)
Others	377 (2.8)
Socio economic position (Obtained from quintiles of SEP score ranging from −4.87 to 6.40)	
Poorest	2572 (19.1)
Second	2808 (20.8)
Middle	2690 (20)
Fourth	2689 (19.99)
Least poor	2692 (20.01)
Living in Delhi >10 yrs	12128 (90.2)
Living in the same locality >5 yrs	11316 (84.3)
Living in the same house >5 yrs	8710 (64.8)
Pregnant women(PW)	N = 594
ᅟMean age (SD)	24.3 (3.84)
ᅟLiterate (%)	503(84.7)
ᅟHomemaker (%)	576 (97)
ᅟMarriage after 18 yrs of age (%)	470 (79.1)
ᅟPrimigravida (%)	202 (34)
ᅟGestation period in months (SD)	5.6 (2.34)
ᅟFirst trimester (%)	140 (23.6)
Recently delivered women (RDM)	N = 596
ᅟMean age (SD)	24.9 (4)
ᅟLiterate (%)	521 (87.4)
ᅟHomemaker (%)	584 (98)
ᅟFirst child (%)	214 (34.9)
ᅟMedian time since childbirth (IQR)	3 (1.8) months

### Prevalence of indicators of reproductive healthcare utilization

Table 3 summarizes the prevalence of the indicators of reproductive healthcare utilization. Among the pregnant women (mean gestational period 5.6 months), 249 (42 %) had an ANC card, out of which 47 % had registered during the first trimester. Three fourth of the registrations (188) were at government run facilities with most of these (125/188) with referral hospitals. Among the RDMs, 70 % had an ANC card, out of which 47 % were registered in the first trimester and 8.4 % registered in their third trimester. Our data showed a higher percentage of ANC registration among RDM as compared to the PW, which could probably be due to delayed ANC registration which was not captured among those PW who were in their early gestational period. Less than half of the mothers (42 %) had ≥4 ANC visits during entire pregnancy and 30 % had not visited the hospital even once during their pregnancy.

**Table 3:** Prevalence of reproductive healthcare utilization among urban poor in Delhi

Indicators	Overall prevalence (%)
	(95 % CI)^a^
ANC registration among pregnant mothers (*n* = 594)	41.9 (37.7, 46.3)
ANC registration among recently delivered mothers (*n* = 596)	70 (64.1, 75.3)
Facility based birthing (*n* = 596)	76.9 (72, 81.1)
ANC visits^b^ (*n* = 596)	
No visits	30 (24.7, 35.9)
Some visit (1–3)	70 (64, 75.2)
ANC visits^b^ (*n* = 596)	
Some visit	57.6 (50.6, 64.2)
≥4 visits	42.4 (35.8, 49.4)
First ANC visit in first trimester (*n* = 417)	46.3 (39.4, 53.3)
Postnatal visit to facility (*n* = 596)	46 (41.5, 50.5)
Possession of Immunization card (*n* = 593)	72.2 (67.5, 76.4)
Any contraceptive use (*n* = 596)	28.2 (21.7, 35.7)
Modern contraceptive use (*n* = 596)	26.3 (20.2, 33.5)

^a^CI computed after taking clustering into account

^b^We present ANC visits in two ways. Some ANC versus None and Some ANC versus Adequate visits. Adequate visits defined as 4 and more visits as per current WHO recommendation

Of the 596 childbirths, 458 (77 %) occurred at a facility, of which three-fourth were public facilities. Of the home deliveries, 80 % were assisted by a traditional birth attendant (*dai*), 70 % used a safe delivery kit, and 21 % were unaware if it was used.

Less than half of the-596 RDM (46 %) had visited a health facility within one month of childbirth, and only half of these had done so within 2 weeks. Only 10 % of these households were visited by a health worker within 2 days of childbirth or discharge from hospital. Five percent of families had received cash under the Janani Suraksha Yojna (JSY) (a conditional cash transfer scheme to promote institutional deliveries), 76 % did not avail the scheme, 3 % reported that they were not entitled, and 16 % were unaware of the scheme. One fourth of RDM (26 %) reported to be using a modern contraceptive method (most commonly condoms).

### Determinants of reproductive healthcare utilization

**Fig. 2:**
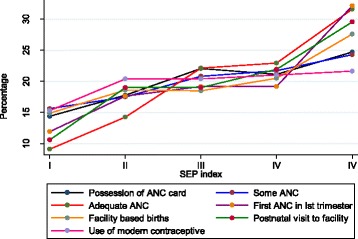
Healthcare utilization indicators by Socio-Economic Position of urban poor households in Delhi

SEP was the single most important determinant for most outcome indicators after adjusting for the other variables (Table 4). Figure 2 shows the trend across the 5 categories of SEP (poorest to least poor) with the prevalence of all indicators being low in the lowest 2 socio-economic quintile. Cluster vulnerability score was associated with registration of ANC within first trimester and facility based birthing with lower odds in women residing in vulnerable clusters. However, the IOR-80 calculated for the vulnerability score included “one”, for the above outcomes, which meant that these factors were not that important in understanding the cluster level variations. Also the outcomes with Median OR larger than 1 (Table 4) indicate the importance of unmeasured cluster level factors in explaining the variations of these reproductive health utilization indicators.

**Table 4:** Determinants of reproductive health care utilization among urban poor

Socio-demographic indicators	Possession of ANC card (*n* = 596 RDM)	Adequate ANC visits (4 and above) (*n* =596 RDM)	Some ANC visit (1 and above) (*n* =596 RDM)	ANC registration in the first trimester (*n* = 417 RDM)	Facility based childbirth (*n* = 596 RDM)	Post-partum visit to health facility (*n* = 596 RDM)	Use of modern contraception (*n* = 596 RDM)
Socio-economic Position							
Poorest (ref)	1	1	1	1	1	1	1
Second	1.13 (0.62, 2.03)	1.22 (0.64, 2.33)	1.13 (0.63, 2.03)	1.20 (0.57, 2.49)	1.18 (0.65, 2.14)	2.16 (1.20, 3.91)	1.19 (0.59, 2.40)
Middle	2.55 (1.35, 4.85)	3.31 (1.75, 6.27)	2.55 (1.35, 4.85)	0.92 (0.45, 1.88)	2.16 (1.13, 4.14)	1.91 (1.05, 3.48)	1.90 (0.93, 3.87)
Fourth	2.65 (1.34, 5.24)	3.83 (1.95, 7.53)	2.65 (1.34, 5.24)	1.00 (0.48, 2.11)	3.89 (1.85, 8.18)	2.76 (1.48, 5.17)	1.82 (0.86, 3.82)
Least poor	1.99 (0.96, 4.15)	5.86 (2.82 12.19)	1.99 (0.96, 4.15)	1.80 (0.81, 3.97)	4.87 (2.12, 11.16)	3.18 (1.62, 6.26)	1.78 (0.78, 4.06)
	0.	<0.001	0.006	0.31	0.0003	0.009	0.35
Cluster vulnerability score (0–10)	1 (0.84, 1.2)	0.85 (0.72, 1.00)	1 (0.84, 1.2)	0.85 (0.72 , 0.99)	0.87 (0.76, 0.99)	1.12 (1.00, 1.26)	0.95 (0.73, 1.23)
	0.98	0.057	0.99	0.041	0.04	0.05	0.68
Interval OR-80	-	0.31- 2.29	-	0.44 - 1.65	0.65 -1.17	1.12-1.12	
Distance of cluster from PUHC (in km)	1.70 (0.68, 4.25)	1.30 (0.56, 3.03)	1.7 (0.68, 4.25)	1.09 (0.48, 2.47)	1.42 (0.68, 2.94)	0.59 (0.32, 1.07)	1.34 (0.37, 4.86)
	0.25	0.55	0.25	0.84	0.35	0.081	0.66
Family size							
Up to 5 members	1	1	1	1	1	1	1
More than 5	1.02 (0.63, 1.67)	0.92 (0.58, 1.45)	1.02 (0.63, 1.67)	1.04 (0.62, 1.76)	0.34 (0.20, 0.59)	0.56 (0.36, 0.88)	1.60 (0.94, 2.72)
	0.93	0.71	0.92	0.88	<0.001	0.012	0.081
Religion							
Non-muslim	1	1	1	1	1	1	1
Muslim	0.95 (0.54, 1.68)	0.85 (0.49, 1.46)	0.95 (0.54, 1.68)	0.91 (0.49, 1.68)	0.44 (0.25, 0.76)	0.88 (0.53, 1.46)	1.11 (0.60, 2.06)
	0.87	0.55	0.87	0.768	0.003	0.623	0.74
Caste							
SC/ST (ref)	1	1	1	1	1	1	1
OBC	0.76 (0.43, 1.32)	1.09 (0.65, 1.84)	0.76 (0.43. 1.32)	1.42 (0.79, 2.56)	1.05 (0.59, 1.86)	1.80 (1.10, 2.94)	0.57 (0.30, 1.08)
General	0.78 (0.49, 1.22)	0.88 (0.57, 1.35)	0.78 (0.50, 1.23)	1.36 (0.84, 2.21)	1.50 (0.92, 2.43)	1.94 (1.30, 2.88)	0.94 (0.58, 1.52)
	0.42	0.66	0.489	0.35	0.23	0. 003	0.20
Family type							
Extended	1	1	1	1	1	1	1
Nuclear	0.66 (0.39, 1.14)	1.71 (1.03, 2.84)	0.67 (0.39, 1.14)	1.22 (0.70, 2.11)	0.60 (0.34, 1.07)	0.46 (0.29, 0.73)	2.28 (1.26, 4.15)
	0.14	0.04	0.14	0.48	0.083	0.001	0.006
Age (3 year interval)	1.12 (0.96, 1.31)	1.1 (0.94, 1.28)	1.12 (0.96, 1.31)	1.04 (0.88, 1.24)	1.14 (0.96, 1.35)	1.14 (1.00, 1.32)	1.12 (0.94. 1.32)
	0.15	0.23	0.16	0.64	0.127	0.065	0.21
Literacy							
Illiterate	1	1	1	1	1	1	1
Literate women	0.57 (0.31, 1.05)	0.80 (0.45, 1.44)	0.57 (0.31, 1.06)	1.10 (0.58, 2.10)	1.23 (0.69, 2.18)	1.43 (0.81, 2.52)	1.07 (0.55, 2.06)
	0.075	0.46	0.075	0.77	0.49	0.21	0.84
Parity							
Multiparous	1	1	1	1	1	1	1
Primi / First child	2.03 (1.29, 3.19)	1.28 (0.85. 1.92)	2.03 ( 1.29, 3.19)	1.35 (0.86, 2.13)	1.68 (1.02, 2.76)	1.65 (1.12, 2.43)	0.70 (0.43, 1.14)
	0.002	0.25	0.002	0.19	0.04	0.011	0.154
ICC (unadjusted), 95 %CI	.084 (0.035, 0.189)	0.126 (0.062 0.238)	0.85 (0.35, 0.189)	0.087 (0.32, 217)	0.09 (0.47, 0.2)	0.011 (0.0002, 0.28)	0.19 (0.11, 0.34)
*P* value for ICC = 0	<0.001	<0.001	<0.001	0.002	<0.001	0.27	<0.001
Model (Variance and SE of	0.13 (0.127)	0.299 (0.1512)	0.360 (0.166)	0.132 (0.1257)	0.0265 (1.004)	1.17e-34 (1.41e-16)	1.05 (0.402)
cluster), Median OR	2.63	1.68	1.77	1.41	1.16	0	2.66

*ANC* Antenatal care, *PW* Pregnant women, *RDM* Recently delivered mothers i.e., in the past 6 months), *PUHC* Primary Urban Health Centre, *SC/ST* Scheduled caste and Scheduled tribe, *OBC* Other Backward class, *ICC* Intra-cluster correlation coefficient

Apart from poverty, other important social determinants for poor health care utilization during pregnancy, childbirth and post-partum period were: lower social class, religion (Muslim), larger households and living in nuclear family, multiparous and younger age of the mother. Primigravida were more likely to avail antenatal care and this was similar with both groups RDM and PW (result of ANC card possession in PW not shown in Table 4). The odds of visiting a health facility after childbirth was twice among those who had registered for ANC (OR 2.14 (95 % CI 1.43, 3.21) and almost three times (OR 2.88 (95 % CI 1.78, 4.67) among those who gave birth in a facility, after adjusting for each other and other socio-demographic factors. (Not shown in Table 4)

**Fig. 3:**
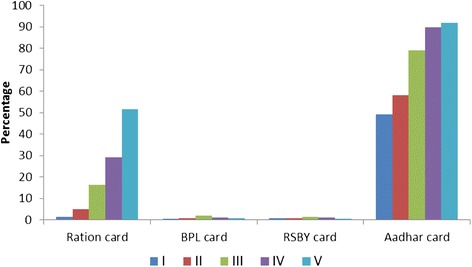
Possession of entitlement cards in % among urban poor households (*n* = 1183) across Socio economic positions (I-V Poorest to Least poor)

Figure 3 presents the proportion of households of PW and RDM (*n* = 1183) in the settlement possessing entitlement cards across SEP quintiles. Most households had Aadhar cards but were very unlikely to have Below Poverty Line (BPL) or health insurance (RSBY) cards. Less poor households were more likely to possess a ration card than poorer ones.

## Discussion

This survey suggests that the utilization of reproductive healthcare services among the urban poor in Delhi is suboptimal. Government benefit schemes like health insurance and conditional cash transfers are also underutilized. Low SEP was the single most important predictor of underutilization with a clear gradient demonstrable across socioeconomic strata.

This survey provides contemporary estimates of the indicators of reproductive healthcare utilization among the urban poor. In comparison with the NFHS-3 (2005–6) which recorded 84.4 % ANC registration among RDM, the proportion was 15 % lower in our study. This may have been due to the difference in the way ANC registration was defined in both surveys. In our study we considered a woman to be registered for ANC only if she had reported possession of an ANC card, whereas in the NFHS survey any ANC care provided by a health worker was considered as ANC [19]. However, the proportion of facility based birthing in our survey was 40 % higher as compared to NFHS-3 (33 % institutional delivery). This increase in facility based birthing since 2005 is encouraging and could be attributed to several factors like increasing awareness and better access to facilities. However, more women belonging to higher SEP tended to give birth at a facility than those belonging to lower SEP (86 vs 57 %). Therefore, it may be possible that much of this increase in facility based birthing may have been caused by a disproportionately greater improvement among women belonging to higher wealth quintiles. This is supported by a recent survey in a poorer slum cluster of Delhi, which showed lower rates of facility-based birthing (48 %), [20] and studies from other developing nations which have demonstrated that the ultra-poor use healthcare facilities the least in urban settings [26, 27, 9, 28]. In any case, even if the rates of institutional delivery have improved since the last national survey, support from the national schemes like JSY or RSBY do not appear to have contributed to this improvement as most of the people in our study did not possess the entitlement cards to these schemes.

SEP was a strong predictor of underutilization of healthcare services. This mirrors the findings from an earlier survey among urban poor in Mumbai [8]. National schemes such as the JSY and RSBY were designed to improve healthcare access to poor households. However, our survey suggests that most poor people do not possess entitlement cards to these schemes. This could be due to inability to produce documentary evidence of being a local resident, being below poverty line or proof of social class. For example non-possession of ration card among the poorest may also indicate social exclusion faced by this migrant population. The widespread availability of the Aadhar card (universal identity card) due to the relentless drive by the government, may facilitate conditional cash transfers through some of these schemes in the future. But for now, the underutilization of health schemes by poorer households highlights the need to identify this “invisible” population and target health interventions to this group as key to achieving UHC [11].

The other socio-demographic determinants of underutilization identified in this study are similar to those in surveys from urban slums in India [1, 20, 8, 22, 23] and other developing nations [2, 29]. Sanneving et al., in a systematic review, showed that economic status, gender, education, social status and age influence the access to maternal and reproductive healthcare in India [29]. Apart from the determinants identified in our study, social factors like low self-esteem and discrimination [9], nomadic living, unfamiliar language and lack of negotiating capacity [30] may also result in poor utilization of reproductive services. Quality of the services offered also has an impact on utilization, in this segment of population [20, 31]. We did not measure these determinants in our survey.

In our study, failing to visit a health facility after child birth was strongly associated lower socio-economic position, larger family size, living in nuclear family, lower caste and multiparity. Distance to the facility was also one of the contributing factors. DHS data from Nepal and Indonesia showed that socio-economic position, literacy, availing antenatal care, facility based birthing and place of residence as important indicators of post-natal visit to health facility [32, 33]. In a systematic review to assess the socioeconomic, geographical and demographic inequities in low- and middle-income countries, socio economic inequality was the most important predictor in the use of postnatal health-care services [34].

In our study population, only one fourth of couples were using contraception (mostly condoms) and living in nuclear families was the single most important predictor. A study in urban slums of Mumbai of family planning in recently delivered mothers [35] also found only 35 % used contraception and non-use was associated with domestic violence. This emphasises the need for counselling of couples on post-partum contraception during postnatal hospital visits or home visits by community health workers.

We noted a large unexplained cluster level variance with most of the outcomes, in spite of adjusting for cluster vulnerability and distance from PUHC indicating unmeasured neighbourhood factors. Clustering of these indicators as evidenced by the ICC’s, suggests that any intervention at a community level is likely to have an impact on individual behaviour.

### Strengths and limitations

The SEP score computed using principal component analysis had good internal consistency and is in general considered a good measure of socioeconomic status [36]. These findings are based on high quality data collected using a validated Electronic Data Capture instrument with strict field quality control.

A non-response rate of 15 % seen in this survey could have been a source of bias if the households that were not available for the survey were socio-demographically different from our final sample of households. Likewise, only women who declared their pregnancy contributed to the analysis. Hence, the estimates obtained for possession of ANC card could be biased if those who disclosed and did not disclose their pregnancy differed significantly. Finally, though we covered all households from two large purposively chosen settlements, this may not be representative of the urban poor in India.

## Conclusion

Our study highlights the need to improve healthcare utilization among the poorest people through a targeted approach. There are several challenges in improving the health of urban poor due to illegal nature of settlements, lack of organized public sector health services in cities, poor coordination between multiple stakeholders, weak linkages between community and service providers, and rural centric policies [30]. Governments should explore the feasibility of risk-profiling of urban poor settlements, households and pregnant women to identify the most vulnerable and linking them to the available national schemes and programs.

Currently link workers, ASHAs are required to conduct a household survey in their respective areas in order to know the community they are serving. Vulnerability of the neighbourhood could be measured by rapid surveys [13]. In order to identify the high risk households in an urban context simple household information like number of rooms, separate kitchen, household assets and other information including household size, family type (nuclear or extended) and religion can be collected. An easy-to-score questionnaire designed for the purpose of risk profiling households, efficient data collection and management systems inbuilt within the health systems, periodic use of data for decision making by the medical officer in-charge can help community health workers in increasing awareness amongst communities and targeting services to the most vulnerable.

## Additional files

Additional file 1:
**Data management of ANCHUL baseline survey.** (DOCX 125 kb) Additional file 2:
**Details of Principle Component Analysis for computing Socio-economic scores.** (DOCX 24 kb)

## Abbreviations

ANC: Antenatal care
PUHC: Primary Urban Healthcare Centre
SEP: Socio-economic Position
PW: Pregnant women
RDM: Recently Delivered Mother
NFHS: National Family Health Survey
ASHA: Accredited Social Health Activist
BPL: Below Poverty Line
RSBY: Rashtriya Swasthya Bima Yojana
JSY: Janani Suraksha Yojna
ICC: Intra cluster Correlation Coefficient
