# Supplementary material for: Reproductive healthcare utilization in urban poor settlements of Delhi: Baseline survey of ANCHUL (Ante Natal and Child Health care in Urban Slums) project
Source: BMC Pregnancy Childbirth. 2015 Sep 8;15:212. doi: 10.1186/s12884-015-0635-8 (PMC4563853; doi:10.1186/s12884-015-0635-8)
Supplement: Additional file 1: — Data management of ANCHUL baseline survey. (DOCX 125 kb) [file 12884_2015_635_MOESM1_ESM.docx]

**Additional file 1: Data management of ANCHUL baseline survey**

**CommCare** (Community Care) is an open source mobile platform designed by Dimagi (http://www.dimagi.com/). CommCare enables easy electronic data collection, decision support, and patient/case management. The mobile application can be used by community health workers and link workers as a job aid as well as a data collection tool. CommCare is multimedia enabled and one can customize audio, video and image prompts. The web application provides reporting, analytics dashboards, user/domain management, and data viewing and analysis.

CommCare consists of two main technology components:

1. CommCareHQ
2. CommCare Mobile

**CommCareHQ**, (<https://www.commcarehq.org/>) is CommCare's cloud based platform for application management (questionnaire or tool designing or development (multiple choice questions, dates, open ended questions) using different languages and images with advanced validation rules i.e. skip order, double entry and range checks) and data access. CommCareHQ receives all data submitted from CommCare Mobile. HQ allows to export all received data in its rawest form into excel or csv. One of the core functionalities of CommCare HQ is its in-built reports that allow project staff and supervisors to monitor link workers. Some of the functionality that HQ reports has are tracking form submissions and time of submission, tracking follow-up rate of beneficiaries by link workers, and also information about the case or beneficiary. Tools designed here are deployed in mobile/tablet either through scanning the bar code or by entering the URL code of application version.

**CommCare Mobile** is a phone or tablet-based client application for data collection and service delivery. It supports two primary mobile platforms: J2ME and ODK-Android. The J2ME or Java application is primarily used on low cost feature phones. The ODK-Android is capable of running on any device (mobile or tablet) that runs on Android OS. Using CommCare, a user can fill out information that gets sent to the server in real time. The advantage of using CommCare is that in situations of low or no connectivity, a user can also work in offline mode and the information gets stored on phone which can be sent when there is connectivity at a later stage.

**Application development for ANCHUL project:** The ODK-Android based application was customized for ANCHUL surveillance tools by the Dimagi team in collaboration with the ANCHUL surveillance team who provided the specifications of the piloted tools. The validation of the app was done as an iterative process until the final version of applications for different modules was finalised before initiating the data collection(Figure 1). The final application had the following modules: Identifier form, Household survey, Participant information, Vital status, Pregnancy, Under 5 child, Death form and Non Interviewer Report Form (NIRF)(Panel 1)


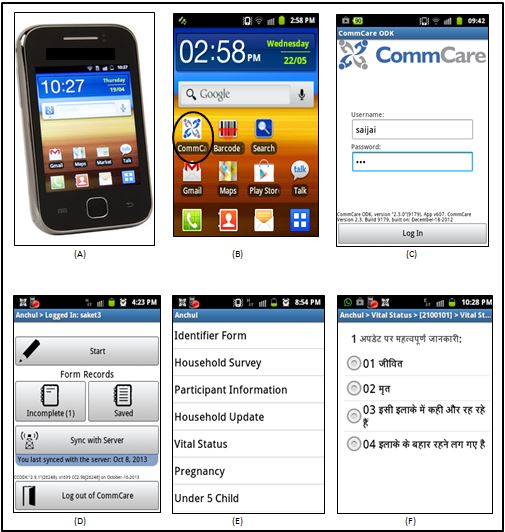
**Panel 1: Images of the mobile appllication**

**(Commcare) of ANCHUL project**

A=Smart phone costing approx. 100USD used for data collection

B=Commcare application after installation in phone

C=Commcare login page

D=Commcare homepage

E=Module page

F=Question page

**Data management team:** The ANCHUL project data management team consisted of Data base administrator, Statistician, Data manager, Surveillance officers, and Field interviewers with predefined roles and responsibilities.

**Training:** Trainings were conducted initially for the trainers by the DIMAGI master trainers and followed by training of filed interviewers/data collectors. Refresher trainings were also conducted on need basis or when a new batch of data collectors were appointed. A field based induction was done whenever a new module was introduced. During the trainings they were briefed about the study procedure, hierarchy of questionnaires, interpretation of each question, methods to ask the question, probable errors, phone setting, battery charging, saving incomplete forms, form editing, sync up with server and do’s or don’ts of phone use. Mock sessions were held before inducting them to field.

**Data collection:** User IDs and mobile phone (without SIM card as we were collecting data offline) were assigned to data collectors. Data collectors needed to login with their respective user ID to collect data. The user ID and the Household IDs were not phone specific but the households that were interviewed by that user ID will open up for further data entry only with entry of the appropriate user login ID. The data collectors collected their fully charged phone from the field office in the morning and deposited the phone after syncing the data with the server using Wi-FI in the evening. Since the mobile phones were not provided with SIM cards it prevented the use of phone for other purposes. Mobile phones were checked on regular basis to confirm if there were incomplete forms or not synced forms. Work report and average time taken/user for data collection could be generated on a daily basis. Data were downloaded and checked for discrepancies on regular basis using STATA 13.

**Figure 1: Flow of activities for data management of surveillance data (ANCHUL project)**

**Quality control measures:**

- Designing
  - Experience trained personnel
  - Documentation
  - SOP’s
- Implementation
  - Trainings and Manuals
  - Re-trainings
  - Random checks at field level
  - 5% recollection for data for selected variables
  - Processing using Statistical software

User Acceptance Testing

Design of questionnaire in EDC using Commcare

Pilot testing of paper forms

Changes in data

Query resolution

Query generation

Post-pilot questionnaire in paper form

Validation of EDC for

- Correctness
- Completeness
- In-built checks
- Output data table
- Coding of data

Freezing of data for further analysis

Training of Field Interviewers

- Administration of questionnaire
- Handling of phone
- Data collection using the application
- Transfer of data

Transferring of data to STATA-13 for processing every fortnightly

Offline data collection in field using smart phone (EDC)

Data transfer to server in filed office after connecting the phone with Wi-Fi internet

Designing of questionnaire in paper forms
