# Supplementary material for: Reproductive healthcare utilization in urban poor settlements of Delhi: Baseline survey of ANCHUL (Ante Natal and Child Health care in Urban Slums) project
Source: BMC Pregnancy Childbirth. 2015 Sep 8;15:212. doi: 10.1186/s12884-015-0635-8 (PMC4563853; doi:10.1186/s12884-015-0635-8)
Supplement: Additional file 2: — Details of Principle Component Analysis for computing Socio-economic scores. (DOCX 24 kb) [file 12884_2015_635_MOESM2_ESM.docx]

**Additional file 2: Details of Principle Component Analysis for computing Socio-economic scores.**

Computing the Socio-Economic Position (SEP): This was derived from dwelling characteristics and household assets using principal component analysis (PCA).([12](file:///D:\IIPHD\Research\MCHN\literature%20search\SES%20HU%20paper\WHO%20submission%20folder\RHC_SEP_24Aug2014.docx#_ENREF_12)) A total of 32 items were initially considered for inclusion of which nine were of dwelling characteristics, 19 items on durable assets and four other variables viz. religion, caste, possession of ration card and below poverty line card. Of these, only the items that had a correlation of |>0.25| with at least three other items were included. Items that showed very high correlation |>0.80| and those that were absent or found in the majority of households were excluded (Table 1, Appendix 1). Using these criteria 18 items were included for deriving the eigenvalue. The predicted SEP score was obtained using only the first component which explained 37% of the variation. The distribution of the score was examined for any obvious clumping or truncation (Figure 1, Appendix 1). This score was divided into quintiles, the lowest representing the poorest and the highest representing the least poor of the study population (reference category). The internal consistency of this scale was checked by tabulating each of the items with the SEP categories (Table 2, Appendix 1).

**Table 1: Variable list and reasons for exclusion for Socio-Economic-Position (SEP) indices using Principal Component Analysis (PCA)**

| S.no | Variable | Group | variable type | Included | Reason for exclusion |
| --- | --- | --- | --- | --- | --- |
| 1 | Religion |  | nominal | No | r< 0.25 |
| 2 | Caste |  | nominal | No | r< 0.25 |
| 3 | Ration card |  | Binary | Yes |  |
| 4 | BPL card |  | nominal | Yes |  |
| 5 | Rented | Dwelling characteristics | Binary | Yes |  |
| 6 | House quality |  | Binary | No | r< 0.25 |
| 7 | Floor type |  | Binary | Yes | All HH |
| 8 | Piped water within HH |  | Binary | No | r< 0.25 |
| 9 | Electricity |  | Binary | Yes | All HH |
| 10 | open defecation |  | Binary | Yes |  |
| 11 | Drainage type |  | categorical | No | r< 0.25 |
| 12 | Number of rooms |  | discrete/grouped | Yes |  |
| 13 | Separate Kitchen |  | Binary | Yes |  |
| 14 | TV | Durable assets | Binary | Yes |  |
| 15 | Refrigerator |  | Binary | Yes |  |
| 16 | Washing machine |  | Binary | Yes |  |
| 17 | Mixer |  | Binary | Yes |  |
| 18 | Air cooler |  | Binary | Yes |  |
| 19 | AC |  | Binary | No | Too few |
| 20 | Room heater |  | Binary | Yes |  |
| 21 | Water heater |  | Binary | Yes |  |
| 22 | Cot |  | Binary | No | Too few |
| 23 | Bore well |  | Binary | No | Too few |
| 24 | number of fans |  | discrete/Grouped | Yes |  |
| 25 | number of cell ph |  | discrete/Grouped | Yes |  |
| 26 | number of Almirahs |  | discrete/Grouped | Yes |  |
| 27 | Number of goats |  | discrete/Grouped | No | Too few |
| 28 | Number of cows |  | discrete/Grouped | No | Too few |
| 29 | Rickshaw |  | Binary | No |  |
| 30 | Bicyle |  | Binary | No | r<0.25 |
| 31 | 2 wheeler(motored) |  | Binary | Yes |  |
| 32 | 4 wheeler (motored) |  | Binary | No | r<0.25 |

**Table 2: Factors included in PCA (distribution in the household, weights assigned for the predicted score and distribution by SEP quintiles to demonstrate internal consistency)**

| Factors | Mean(SD) | Weights | Mean by quintiles | | | | |
| --- | --- | --- | --- | --- | --- | --- | --- |
|  |  |  | Least poor | Fourth | Middle | Second | Poorest |
| Rented  Yes  No (ref) | 0.40(0.49)  0.60(0.49) | -0.23 | 0.03 | 0.14 | 0.37 | 0.64 | 0.81 |
| Ration card  Yes  No (ref) | 0.27 (0.44)  0.72(0.44) | 0.15 | 0.48 | 0.40 | 0.27 | 0.13 | 0.05 |
| Durable housing  Yes (ref)  No | 0.84 (0.37)  0.16(0.37) | -0.13 | 0.02 | 0.06 | 0.14 | 0.20 | 0.40 |
| Open defecation  Yes(ref)  No | 0.12 (0.33)  0.88 (0.33) | -0.12 | 0.003 | 0.05 | 0.12 | 0.16 | 0.30 |
| Rooms | 1.62(0.83) | 0.29 | 2.7 | 1.9 | 1.4 | 1.1 | 1 |
| Separate Kitchen  Yes (ref)  No | 0.53(0.5)  0.47(0.5) | -0.26 | 0.05 | 0.17 | 0.44 | 0.75 | 0.96 |
| Fans | 1.48(0.71) | 0.29 | 2.4 | 1.7 | 1.2 | 1.03 | 0.96 |
| Almirahs | 0.51 (0.58) | 0.30 | 1.2 | 0.8 | 0.4 | 0.1 | 0.01 |
| Cell phones | 1.70 (0.76) | 0.23 | 2.4 | 1.9 | 1.7 | 1.4 | 1.1 |
| TV  Yes(ref)  No | 0.88(0.33)  0.12(0.33) | -0.17 | 0.001 | 0.003 | 0.025 | 0.083 | 0.52 |
| Refrigerator  Yes(ref)  No | 0.59(0.49)  0.41 (0.49) | -0.28 | 0.005 | 0.047 | 0.29 | 0.74 | 0.98 |
| Washing machine  Yes  No(ref) | 0.23(0.42)  0.77(0.42) | 0.25 | 0.78 | 0.27 | 0.08 | 0.01 | 0.001 |
| Mixer  Yes  No(ref) | 0.24(0.43)  0.76(0.43) | 0.25 | 0.78 | 0.32 | 0.09 | 0.02 | 0.007 |
| Air-cooler  Yes  No(ref) | 0.52(0.49)  0.48(0.49) | 0.26 | 0.95 | 0.79 | 0.57 | 0.25 | 0.02 |
| Room heater  Yes  No(ref) | 0.04(0.19)  0.96(0.19) | 0.13 | 0.17 | 0.025 | 0.004 | 0.001 | 0 |
| Water heater  Yes  No(ref) | 0.33(0.47)  0.77(0.47) | 0.21 | 0.74 | 0.47 | 0.27 | 0.16 | 0.02 |
| Motor cycle  Yes  No(ref) | 0.31(0.46)  0.69(0.46) | 0.24 | 0.79 | 0.45 | 0.19 | 0.08 | 0.01 |

**Fig 1: Spread of the predicted SEP score to show clumping or truncation**
